# Clinical models for predicting 30-day mortality in ARDS: A focus on ventilatory ratio-defined subgroups

**DOI:** 10.1016/j.jointm.2025.09.002

**Published:** 2025-12-08

**Authors:** Zhangwei Liang, Xinyi Luo, Ya Wang, Weilin Wang, Yuelin Liu, Ying Zhu, Juan Ouyang, Jie Zhang, Yin Xi, Yimin Li, Yonghao Xu

**Affiliations:** Department of Critical Care Medicine, The First Affiliated Hospital of Guangzhou Medical University, State Key Laboratory of Respiratory Disease, National Clinical Research Center for Respiratory Disease, National Center for Respiratory Medicine, Guangzhou Institute of Respiratory Health, Guangzhou, Guangdong, China

**Keywords:** Acute respiratory distress syndrome, Database, Mortality, Prediction, Ventilatory ratio

## Abstract

**Background:**

It is supposed that acute respiratory distress syndrome (ARDS) patients with increased dead space, indicated by elevated ventilatory ratio (VR), had higher mortality. The difference in mortality predictors among ARDS patients categorized by VR remains unclear, so we aimed to investigate the risk factors for mortality prediction in subgroups defined by VR and develop risk models to predict the 30-day mortality in distinct ARDS subgroups.

**Methods:**

This study performed a retrospective analysis using the Medical Information Mart for Intensive Care IV (MIMIC-IV) and eICU Collaborative Research Database (eICU-CRD) databases, as well as data from NHLBI ARDS Clinical Trials Network (ARDSnet). Patients were divided into high VR (VR ≥2) and low VR (VR <2) subgroups based on baseline VR. In ARDSnet cohort, two 30-day mortality risk prediction models were constructed using logistic regression, internally validated using the bootstrap method, and externally validated in the MIMIC-IV and eICU-CRD cohorts. The performance of models was evaluated using receiver operating characteristic curves, Brier scores, calibration curves, and decision curve analysis (DCA) curves, and DeLong test was used to compare the predictive efficacy of different models in each subgroup.

**Results:**

This study included a total of 2977 ARDS patients: 1031 in the high VR training cohort, 1506 in the low VR training cohort, 159 in the high VR external validation cohort, and 281 in the low VR external validation cohort. Through stepwise regression analysis, 11 predictors were finally selected to construct high VR prediction model including age, body mass index (BMI), heart rate, mean arterial pressure, body temperature, blood urea nitrogen (BUN), bilirubin, minute ventilation, peak inspiratory pressure, inspired oxygen fraction (FiO_2_), and peripheral oxygen saturation (SpO_2_), and 13 predictors to construct low VR prediction model including age, heart rate, body temperature, platelet count, BUN, bilirubin, respiratory rate, peak inspiratory pressure, FiO_2_, hypercapnia, hypocapnia, acidemia, and alkalemia. The area under the curve (AUC) of high VR model in the training cohort was 0.76 (95 % CI: 0.73 to 0.79), and the AUC of low VR model in training cohort was 0.76 (95 % CI: 0.74 to 0.79). The Brier scores for high VR model and low VR model in training cohort were 0.171 and 0.139, respectively. Decision curve analysis (DCA) showed that the DCA curve for high VR model waspositioned away from two extreme curves across a threshold probability range of 0.2 to 0.8, and the curve for low VR model was positioned away from two extreme curves across a threshold probability range of 0.1 to 0.6. The AUC of high VR model in low VR subgroup training set was 0.74, significantly lower than the low VR model (DeLong test, *P*=0.024). The AUC of low VR model in high VR subgroup training set was 0.73, significantly lower than high VR model (DeLong test, *P*=0.001).

**Conclusion:**

This study developed and validated prognostic prediction models for patients of high and low VR subgroups, respectively. The models demonstrated good discrimination, calibration, and clinical utility. Prognostic risk factors differed between high and low VR subgroups, and prediction models developed for specific VR subgroups exhibited better predictive performance within their respective subgroup populations.

## Introduction

Acute respiratory distress syndrome (ARDS) is a threatening syndrome characterized by increased alveolar-capillary membrane permeability, increased non-aerated lung tissue, leading to increased venous admixture and dead space ventilation.^[^^1^^]^ ARDS is a heterogeneous clinical syndrome, and this heterogeneity might explain the limited effectiveness of interventions in randomized controlled trials (RCTs) of treatments.^[^^2^^,^^3^^]^ In response, researchers have identified ARDS subphenotypes with differing prognoses and treatment responses.^[^[4], [5], [6]^]^ For instance, secondary analysis of the ALVEOLI trial identified hypo-inflammatory and hyper-inflammatory sub-phenotypes that responded differently to levels of positive end-expiratory pressure (PEEP).^[^^4^^]^ Similarly, the LIVE trial identified radiographic subphenotypes (focal *vs.* diffuse), and a personalized approach to ventilation based on these findings reduced 90-day mortality in a per-protocol analysis.^[^^7^^]^ These efforts support the use of physiological or biological stratification to advance precision medicine in ARDS.

Despite these advances, current ARDS stratification largely relies on oxygenation indices such as the ratio of arterial oxygen partial pressure to inspired oxygen fraction (PaO_2_/FiO_2_), which do not reflect ventilatory inefficiency.^[^^1^^,^^8^^]^ This is a critical omission, as impaired carbon dioxide clearance due to increased dead space is also a strong, independent predictor of mortality in ARDS.^[^^9^^]^ Moreover, the heterogeneity of ARDS continues to challenge the development of broadly effective therapies.^[^^10^^]^ In this context, large-scale, real-world databases such as Medical Information Mart for Intensive Care IV (MIMIC-IV) and eICU Collaborative Research Database (eICU-CRD) have become essential resources for exploring this complexity. These datasets allow researchers to identify novel prognostic factors, define clinical subgroups, and construct predictive models tailored to specific phenotypes.^[^^11^^,^^12^^]^ Subgroup-specific nomograms and risk scores derived from such databases have already demonstrated clinical value in guiding individualized care decisions.

Pulmonary dead space fraction (VD/VT) is a well-established predictor of mortality in ARDS.^[^^13^^,^^14^^]^ However, direct measurement of VD/VT requires volumetric capnography or indirect calorimetry, limiting its widespread clinical application. This gap has led to increasing interest in surrogate bedside indices that can approximate dead space ventilation more practically. The ventilatory ratio (VR) has recently emerged as a simple, bedside-calculable surrogate for physiological dead space.^[^^15^^]^ VR incorporates routinely available respiratory variables and has demonstrated a positive correlation with pulmonary dead space. Prior studies have shown that elevated VR is associated with increased mortality in ARDS, and that patients with VR ≥2 have significantly worse outcomes than those with lower values.^[^^16^^]^ Moreover, VR thresholds above 2.5–3 have been used as criteria for initiating advanced support therapies such as extracorporeal carbon dioxide removal (ECCO_2_R) to facilitate ultra-protective ventilation strategies.^[^^17^^]^ The threshold of VR ≥2 used in our study is based on prior evidence suggesting a meaningful distinction in mortality risk at this value^[^^16^^]^ and reflects a clinically relevant turning point in ventilatory efficiency.

In this study, we aimed to improve ARDS risk stratification by leveraging the VR as a physiologic marker of ventilatory inefficiency. Using data from four NHLBI ARDS Network trials, MIMIC-IV, and eICU-CRD, we developed subgroup-specific models to predict 30-day mortality and assessed the short-term stability of VR classification. By identifying distinct risk factors within each group, this approach supports a more individualized framework for prognostication and ventilation management in ARDS, addressing the physiological heterogeneity.

## Methods

### Data source

We used NHLBI-sponsored ARDS Network RCTs, the MIMIC-IV (version 2.2), and the eICU-CRD to enroll ARDS patients. We enrolled ARDS patients from the ALVEOLI study, the ARMA/KARMA/LARMA study, the ALTA study, and the FACCT study within the NHLBI ARDS Clinical Trials Network (ARDSnet) as model development cohorts. Patients from the MIMIC-IV and eICU-CRD databases were enrolled as external validation cohorts. Certificate number 54892932 was issued to Zhangwei Liang for the use of the MIMIC-IV and eICU-CRD databases. Ethical approval for the databases was obtained from the Institutional Review Boards (IRBs) at Beth Israel Deaconess Medical Center (BIDMC) and the Massachusetts Institute of Technology (MIT). Since the databases do not contain identified health information, a waiver of informed consent was included in the approval. This study was completed before the ARDSnet organization requested the termination of the research and the cessation of the use of its data.

### Study population

Patients included in the study from the MIMIC-IV and eICU-CRD databases met the following criteria: (1) patients aged 16 years or older; (2) patients diagnosed with ARDS within the first 48 h of ventilation; (3) patients receiving invasive ventilation for at least 48 consecutive hours; and (4) patients admitted to the ICU between 2014 and 2019. Patients meeting the following criteria were excluded from the study: (1) patients readmitted to the ICU; (2) patients receiving ventilation through a tracheostomy tube; (3) patients receiving extracorporeal membrane oxygenation (ECMO) support during mechanical ventilation; (4) patients with a diagnosis of asthma, chronic obstructive pulmonary disease (COPD), pulmonary hypertension, or pulmonary embolism; (5) lack of baseline VR records.

Patients included in the study from the ARDSnet met the following criteria: patients aged 16 years or older. Patients meeting the following criteria were excluded from the study. The exclusion criteria were: (1) patients receiving ventilation through a tracheostomy tube; (2) patients receiving ECMO support during mechanical ventilation; and (3) lack of baseline VR records.

### Data collection and formula

Demographic data, vital signs, and ventilation parameters were collected on the first day of ventilation in the MIMIC- IV and eICU-CRD databases, and on the first day of enrollment in the ARDSnet. Laboratory values were recorded at the first measurement after ventilation in the MIMIC-IV and eICU- CRD databases, and the most recent values before randomization were captured in the ARDSnet. Vital signs and ventilation parameters were captured as the most recent values around the blood gas record. Data regarding 30-day mortality were also extracted. According to Sinha et al.^[^^16^^]^, VR was estimated using the following formula, where PBW represents predicted body weight and V·E represents minute ventilation:

$$VR=\frac{\dot{V}E_{measured}\left(ml/min\right)\;\times PaCO_{2}\left(mmHg\right)}{100\;\times PBW\left(kg\right)\;\times \;37.5}.$$

### Statistical analysis

Patients were classified into two distinct subgroups based on baseline VR values. ARDS patients with VR values ≥ 2 were defined as high VR subgroup, while those with VR values <2 were defined as low VR subgroup. This study used multivariable logistic regression to develop predictive models for the prognosis of each subgroup separately. Kaplan–Meier survival curves were plotted, and a log-rank test was used to compare survival curves. Univariable logistic regression combined with stepwise logistic regression was used to select variables of models. The variables with *P* values <0.1 in univariable logistic regression would be selected for stepwise regression, and the variables in the optimal subset of the stepwise regression with *P* values <0.05 were selected for the construction of final models. To evaluate the discriminating ability of the models, we calculated the area under the receiver operating characteristic curve (AUROC) of models. Calibration curves and Brier scores were constructed for calibration evaluation. We used decision curve analysis (DCA) to determine the clinical usefulness of our models. Receiver operating characteristic curves and calibration curves were plotted for internal validation of our models using bootstrapping technique. The relative importance of variables for both models was measured as the partial chi-square statistic minus the predictor degrees of freedom.

For continuous variables, according to their distributional characteristics, normally distributed data were presented as mean ± standard deviation (SD), while non-normally distributed data were presented as median and interquartile range (IQR). The distribution of normally distributed continuous variables was verified using the Kolmogorov–Smirnov test. Depending on the distribution of the data and the number of comparison groups, Student’s *t*-test, one-way ANOVA, Mann–Whitney *U* test, or Kruskal–Wallis *H*-test was used to compare differences in continuous variables. Categorical variables were presented as frequencies and percentages and were assessed using the chi-squared (*χ*^*2*^) test or Fisher’s exact test, as appropriate, depending on the sample size. Variables with >30 % missing data were excluded from the variable screening process. Missing data for variables with <30 % missing in the model development cohort were imputed using the multivariate imputation by chained equations (MICE) package, while imputation was not conducted for the validation cohorts. R, version 3.3.2 (R Foundation for Statistical Computing, Vienna, Austria) was used for all analyses. A two-tailed test was performed and *P* <0.05 was considered statistically significant.

## Results

### Participants and the characteristics of the final cohorts

**Figure 1:**
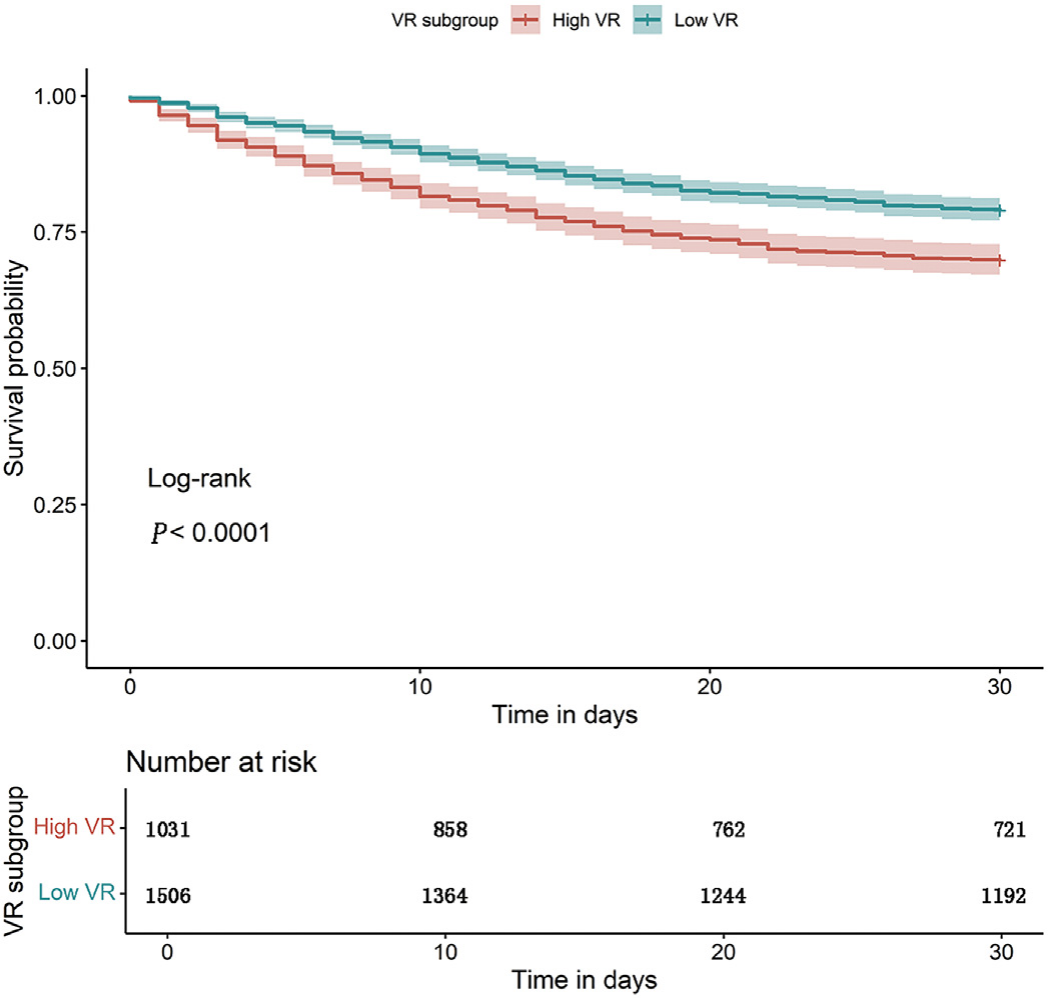
Kaplan–Meier survival curves for patients with VR below 2 *vs.* those with VR above or equal to 2. The outcome measured was 30-day survival. VR: Ventilatory ratio.

Within the ARDSnet cohort, 2537 patients were included in the final analysis. Among these, 1031 patients were allocated to high VR subgroup model development cohort, and 1506 patients were allocated to low VR subgroup model development cohort. Compared to low VR subgroup, patients in high VR subgroup had statistically different probability of survival at Day 30 (P < 0.0001; Figure 1). They also exhibited lower PaO₂/FiO₂ ratios, higher PaCO₂ levels, elevated driving pressures, and reduced static compliance (Table 1).

**Table 1:** Baseline characteristics categorized by baseline VR.

Characteristics	High-VR group (*n*=1031)	Low-VR group (*n*=1506)	*P* value
Age (years)	49.00 (39.00–61.00)	50.00 (38.00–64.00)	0.051
Sex			<0.001
Male	497 (48.2)	921 (61.2)	
Female	534 (51.8)	585 (38.8)	
Ethnicity			0.224
White	733 (71.1)	1027 (68.2)	
Black	183 (17.7)	281 (18.7)	
Other	115 (11.2)	198 (13.1)	
BMI (kg/m^2^)	27.00 (22.81–32.47)	26.50 (22.70–30.49)	0.003
ARDS risk factors			
Pneumonia	482 (46.8)	533 (35.4)	<0.001
Sepsis	249 (24.2)	369 (24.5)	0.877
Aspiration	130 (12.6)	256 (17.0)	0.003
Trauma	56 (5.4)	163 (10.8)	<0.001
Other	114 (11.1)	185 (12.3)	0.38
Laboratory values			
White blood cell (× 10^9^/L)	12.00 (7.23–17.15)	11.70 (7.80–16.50)	0.594
Platelets (× 10^9^/L)	167.0 (92.0–253.5)	152.0 (90.0–236.0)	0.028
HCT (%)	30.0 (27.0–34.0)	30.0 (27.0–34.0)	0.620
PaO_2_ (mmHg)	75.0 (65.0–92.5)	80.0 (69.0–99.0)	<0.001
PaCO_2_ (mmHg)	41.0 (36.0–47.0)	36.0 (31.0–41.0)	<0.001
pH	7.36 (7.29–7.42)	7.41 (7.35–7.45)	<0.001
PaO_2_/FiO_2_ ratio (mmHg)	116.67 (86.83–164.64)	154.00 (111.67–204.72)	<0.001
Bicarbonate (mmol/L)	22.0 (19.0–26.0)	22.0 (19.0–26.0)	0.314
BUN (mg/dL)	19.0 (13.0–33.0)	19.0 (12.0–33.0)	0.939
Creatinine (mg/dL)	1.00 (0.70–1.70)	1.10 (0.80–1.70)	0.005
Bilirubin (mg/dL)	0.90 (0.50–1.80)	0.90 (0.50–1.70)	0.858
Ventilator parameters			
Tidal volume/PBW (mL/kg)	7.78 (6.40–9.74)	8.00 (6.56–9.75)	0.081
PEEP (cmH_2_O)	10 (7–12)	8 (5–10)	<0.001
Plateau pressure (cmH_2_O)	29.00 (25.00–34.00)	25.00 (21.00–30.00)	<0.001
Peak inspiratory pressure (cmH_2_O)	35.00 (30.00–42.00)	32.00 (26.00–38.00)	<0.001
Driving pressure (cmH_2_O)	19.00 (15.00–24.00)	17.00 (13.00–21.00)	<0.001
Compliance (mL/cmH_2_O)	26.19 (20.29–34.21)	33.00 (25.43–42.70)	<0.001
FiO_2_ (%)	70.0 (50.0–90.0)	55.0 (40.0–70.0)	<0.001
Respiratory rate (breaths/min)	28 (22–32)	19 (15–24)	<0.001
Minute ventilation (L/min)	14.3 (12.0–16.9)	10.4 (8.6–12.6)	<0.001
Ventilatory ratio	2.44 (2.19–2.89)	1.55 (1.31–1.76)	<0.001
Vital signs			
Heart rate (beats/min)	107.14 ±20.25	99.24 ±20.04	<0.001
MAP (mmHg)	75.0 (67.0–85.0)	76.0 (69.0–87.0)	0.003
Body temperature (°C)	37.67±1.03	37.51±1.01	<0.001
SpO_2_ (%)	95.0 (92.0–97.0)	96.0 (94.0–98.0)	<0.001
30-Day mortality	311 (30.2)	317 (21.0)	<0.001

Data are presented as mean ± standard deviation for normally distributed variables, median (interquartile range) for non-normally distributed variables, and *n* (%) for categorical variables.

ARDS: Acute respiratory distress syndrome; BMI: Body mass index; BUN: Blood urea nitrogen; FiO_2_: Inspired oxygen fraction; HCT: Hematocrit; MAP: Mean arterial pressure; PaO_2_: Partial pressure of oxygen; PaCO_2_: Partial pressure of carbon dioxide; PEEP: Positive end-expiratory pressure; SpO_2_: Peripheral oxygen saturation; VR: Ventilatory ratio.

A total of 159 patients from the MIMIC-IV and eICU-CRD databases were assigned to the external validation cohort for the high VR subgroup model, and 281 patients for the low VR subgroup model (Supplementary Figure S1). In the development cohort of high VR model, 311 patients (30.2 %) died within 30 days of enrollment (Table 2). In the low VR model development cohort, 317 patients (21.0 %) died within 30 days of enrollment (Table 3). Demographic characteristics, vital signs, laboratory results, and mechanical ventilation settings of the external validation cohorts for the high and low VR subgroups are summarized in Supplementary Table S1.

**Table 2:** Baseline and clinical characteristics of patients categorized into high VR subgroup.

Characteristics	Survivors (*n*=720)	Non-survivors (*n*=311)	*P* value
Age (years)	46.00 (37.00–57.00)	57.00 (43.00–70.00)	<0.001
Sex			0.151
Male	336 (46.7)	161 (51.8)	
Female	384 (53.3)	150 (48.2)	
Ethnicity			0.04
White	528 (73.3)	205 (65.9)	
Black	121 (16.8)	62 (19.9)	
Other	71 (9.9)	44 (14.1)	
BMI (kg/m^2^)	27.50 (23.10–32.66)	25.86 (22.20–31.33)	0.005
ARDS risk factors			
Pneumonia	334 (46.4)	148 (47.6)	0.775
Sepsis	165 (22.9)	84 (27.0)	0.183
Aspiration	94 (13.1)	36 (11.6)	0.579
Trauma	49 (6.8)	7 (2.3)	0.005
Other	78 (10.8)	36 (11.6)	0.81
Laboratory values			
White cell count (× 10^9^/L)	12.00 (7.60–17.10)	12.00 (6.55–17.55)	0.57
Platelets (× 10^9^/L)	174.50 (104.75–257.50)	145.00 (72.50–241.00)	0.001
HCT (%)	30.0 (27.0–34.0)	30.0 (27.0–33.0)	0.439
PaO_2_ (mmHg)	76.0 (66.00–93.0)	73.0 (64.00–91.0)	0.08
PaCO_2_ (mmHg)	41.00 (36.00–47.00)	39.0 (34.0–46.0)	0.05
pH	7.37 (7.30–7.42)	7.35 (7.26–7.40)	<0.001
PaO_2_/FiO_2_ ratio (mmHg)	122.50 (90.00–167.62)	106.67 (80.00–150.00)	<0.001
Bicarbonate (mmol/L)	23.0 (20.0–26.0)	21.0 (18.0–25.0)	<0.001
BUN (mg/dL)	17.0 (11.0–27.0)	26.0 (16.0–45.0)	<0.001
Creatinine (mg/dL)	0.90 (0.70–1.42)	1.30 (0.90–2.10)	<0.001
Bilirubin (mg/dL)	0.80 (0.50–1.60)	1.00 (0.50–2.30)	0.002
Ventilator parameters			
Tidal volume/PBW (mL/kg)	7.80 (6.40–9.71)	7.96 (6.57–10.04)	0.329
PEEP (cmH_2_O)	10 (7–12)	10 (8–13)	0.048
Plateau pressure (cmH_2_O)	28.0 (24.0–33.0)	30.0 (26.0–35.5)	0.002
Peak inspiratory pressure (cmH_2_O)	35.00 (30.00–40.00)	37.00 (30.00–43.50)	0.009
Driving pressure (cmH_2_O)	18.00 (14.00–23.00)	20.00 (16.00–24.25)	0.004
Compliance (mL/cmH_2_O)	26.92 (20.56–34.58)	25.00 (20.00–34.11)	0.202
FiO_2_ (%)	60.0 (50.0–80.0)	70.00 (60.0–92.0)	<0.001
Respiratory rate (breaths/min)	27 (22–32)	28 (22–34)	0.1
Minute ventilation (L/min)	14.0 (11.6–16.3)	15.2 (12.4–18.0)	<0.001
Ventilatory ratio	2.40 (2.17–2.84)	2.52 (2.26–3.00)	0.001
Vital signs			
Heart rate (beats/min)	106.28±19.69	109.12±21.41	0.039
MAP (mmHg)	76.0 (69.0–87.0)	72.0 (65.0–81.5)	<0.001
Body temperature (°C)	37.73±0.92	37.60±1.01	0.039
SpO_2_ (%)	95.0 (93.0–97.0)	94.0 (91.0–97.0)	<0.001

Data are presented as mean ± standard deviation for normally distributed variables, median (interquartile range) for non-normally distributed variables, and *n* (%) for categorical variables.

ARDS: Acute respiratory distress syndrome; BMI: Body mass index; BUN: Blood urea nitrogen; FiO_2_: Inspired oxygen fraction; HCT: Hematocrit; MAP: Mean arterial pressure; PaO_2_: Partial pressure of oxygen; PaCO_2_: Partial pressure of carbon dioxide; PEEP: Positive end-expiratory pressure; SpO_2_: Peripheral oxygen saturation; VR: Ventilatory ratio.

**Table 3:** Baseline and clinical characteristics of patients categorized into low VR subgroup.

Characteristics	Survivors (*n*=1189)	Non-survivors (*n*=317)	*P* value
Age (years)	49.00 (37.00–61.00)	60.00 (46.00–72.00)	<0.001
Sex			0.263
Male	718 (60.4)	203 (64.0)	
Female	471 (39.6)	114 (36.0)	
Ethnicity			0.078
White	827 (69.6)	200 (63.1)	
Black	210 (17.7)	71 (22.4)	
Other	152 (12.8)	46 (14.5)	
BMI (kg/m^2^)	26.75 (22.78–30.85)	25.72 (22.25–29.44)	0.014
ARDS risk factors			
Pneumonia	429 (36.1)	104 (32.8)	0.309
Sepsis	262 (22.0)	107 (33.8)	<0.001
Aspiration	203 (17.1)	53 (16.7)	0.948
Trauma	150 (12.6)	13 (4.1)	<0.001
Other	145 (12.2)	40 (12.6)	0.914
Laboratory values			
White cell count (× 10^9^/L)	11.70 (8.00–16.40)	11.40 (6.40–16.70)	0.05
Platelets (× 10^9^/L)	157.00 (94.00–240.00)	131.00 (65.00–204.00)	<0.001
HCT (%)	30.0 (27.0–34.0)	30.0 (26.0–34.0)	0.15
PaO_2_ (mmHg)	81.0 (70.0–99.0)	78.0 (66.0–98.0)	0.029
PaCO_2_ (mmHg)	37.0 (31.0–41.0)	34.0 (29.0–38.0)	<0.001
pH	7.41 (7.36–7.45)	7.39 (7.32–7.45)	0.001
PaO_2_/FiO_2_ ratio (mmHg)	158.00 (116.67–208.00)	138.18 (99.00–190.00)	<0.001
Bicarbonate (mmol/L)	23.0 (19.0–26.0)	20.0 (17.0–24.0)	<0.001
BUN (mg/dL)	18.0 (12.0–31.0)	26.0 (16.0–42.0)	<0.001
Creatinine (mg/dL)	1.00 (0.80–1.60)	1.40 (0.90–2.10)	<0.001
Bilirubin (mg/dL)	0.80 (0.50–1.50)	1.20 (0.60–2.46)	<0.001
Ventilatior parameters			
Tidal volume/PBW (mL/kg)	8.02 (6.66–9.71)	8.22 (6.80–10.04)	0.207
PEEP (cmH_2_O)	8 (5–10)	8 (5–10)	0.365
Plateau pressure (cmH_2_O)	25.0 (21.0–30.0)	26.0 (21.0–31.0)	0.061
Peak inspiratory pressure (cmH_2_O)	31.00 (26.00–37.00)	33.00 (26.00–38.00)	0.046
Driving pressure (cmH_2_O)	16.00 (12.00–20.00)	17.00 (13.00–21.00)	0.028
Compliance (mL/cmH_2_O)	33.13 (26.09–42.86)	32.43 (25.00–41.92)	0.390
FiO_2_ (%)	50.0 (40.0–70.0)	60.0 (50.0–80.0)	<0.001
Respiratory rate (breaths/min)	19 (15–24)	20 (16–26)	<0.001
Minute ventilation (L/min)	10.2 (8.4–12.2)	11.2 (9.2–13.8)	<0.001
Ventilatory ratio	1.55 (1.31–1.76)	1.59 (1.34–1.80)	0.077
Vital signs			
Heart rate (beats/min)	98.27±19.46	102.95±21.71	<0.001
MAP (mmHg)	77.0 (69.0–88.0)	74.0 (66.0–84.0)	<0.001
Body temperature (°C)	37.54±1.00	37.38±1.02	0.011
SpO_2_ (%)	96.0 (94.0–98.0)	95.0 (93.0–98.0)	0.003

Data are presented as mean ± standard deviation for normally distributed variables, median (interquartile range) for non-normally distributed variables, and *n* (%) for categorical variables.

ARDS: Acute respiratory distress syndrome; BMI: Body mass index; BUN: Blood urea nitrogen; FiO_2_: Inspired oxygen fraction; HCT: Hematocrit; MAP: Mean arterial pressure; PaO_2_: Partial pressure of oxygen; PaCO_2_: Partial pressure of carbon dioxide; PEEP: Positive end-expiratory pressure; SpO_2_: Peripheral oxygen saturation; VR: Ventilatory ratio.

### Stability of VR subgroups from day 0 to day 1

To assess the stability of VR subgroup assignments, we analyzed patient transitions between the high VR and low VR subgroups from Day 0 to Day 1. Among patients initially classified into the high VR subgroup on Day 0, the majority, 694 patients (89.8 %), remained in the high VR subgroup on Day 1, while 79 patients (10.2 %) transitioned to the low VR subgroup. Similarly, for patients initially in the low VR subgroup on Day 0, 1012 patients (94.2 %) maintained their low VR subgroup assignment on Day 1, with only 62 patients (5.8 %) transitioning to the high VR subgroup (Supplementary Table S2).

### Predictor selection and model development

In univariable logistic regression analysis for high VR subgroup, a total of 22 variables were screened for inclusion in the stepwise regression model. These variables included: age, body mass index (BMI), heart rate, mean arterial pressure (MAP), body temperature, SpO_2_, platelet count, serum creatinine, blood urea nitrogen (BUN), bilirubin, respiratory rate, minute ventilation, PEEP, plateau pressure, peak inspiratory pressure, FiO_2_, PaO_2_/FiO_2_ ratio, bicarbonate, hypercapnia, hypocapnia, acidemia, and alkalemia (Supplementary Table S3). Similarly, for the low VR subgroup, univariable logistic regression analysis also identified 24 variables as candidates for stepwise regression. These variables were: age, BMI, heart rate, MAP, body temperature, SpO_2_, white blood cell count, platelet count, serum creatinine, BUN, bilirubin, tidal volume, respiratory rate, minute ventilation, plateau pressure, peak inspiratory pressure, driving pressure, FiO_2_, PaO_2_/FiO_2_ ratio, bicarbonate, hypercapnia, hypocapnia, acidemia, and alkalemia (Supplementary Table S4).

Variables selected from univariable regression analysis were then entered into multivariable logistic regression analysis. Bidirectional stepwise regression was employed for variable selection. Subsequently, variables with a *P* value <0.05 in the optimal subset identified by stepwise regression were included in a final multivariate logistic regression model to determine the final predictive model. The final prognostic risk factors identified for the high VR subgroup included: age, BMI, heart rate, MAP, body temperature, BUN, bilirubin, minute ventilation, peak inspiratory pressure, FiO_2_, and SpO_2_. For the low VR subgroup, the identified prognostic risk factors were age, heart rate, body temperature, platelet count, BUN, total bilirubin, respiratory rate, peak inspiratory pressure, FiO_2_, hypercapnia, hypocapnia, acidemia, and alkalemia (Table 4). Among these, hypocapnia and acidemia were significant risk factors for the low VR subgroup, and alkalemia showed a near-significant association with 30-day mortality, while hypercapnia was not a significant risk factor for the low VR subgroup. Collinearity analysis was conducted for these factors, and no significant collinearity was detected among the variables (Supplementary Figure S2). Supplementary Figures S3 and S4 present the nomograms of our models.

**Table 4:** Multivariable logistic regression models for predicting 30-day mortality.

Variables	Odd ratio	95 % CI	*P* value
High VR model			
Age	1.05	1.03–1.06	<0.001
BMI	0.98	0.96–1.00	0.026
Heart rate(per 10 beats/min)	1.13	1.05–1.23	0.002
MAP (per 10mmHg)	0.86	0.77–0.97	0.011
Body temperature	0.82	0.69–0.96	0.016
BUN	1.01	1.01–1.02	<0.001
Bilirubin	1.06	1.01–1.13	0.033
Minute ventilation	1.06	1.02–1.10	0.002
Peak inspiratory pressure	1.02	1.01–1.04	0.002
FiO_2_ (per 5 %)	1.06	1.02–1.10	0.005
SpO_2_	0.96	0.93–0.99	0.025
Low VR model			
Age	1.05	1.04–1.06	<0.001
Heart rate (per 10 beats/min)	1.26	1.16–1.36	<0.001
Body temperature	0.84	0.74–0.96	0.013
Platelets (per 10 × 10^9^/L)	0.98	0.97–0.99	0.007
BUN	1.01	1.00–1.01	0.021
Bilirubin	1.08	1.04–1.13	0.002
Respiratory rate (per 5 breaths/min)	1.14	1.02–1.26	0.015
Peak inspiratory pressure	1.03	1.01–1.04	<0.001
FiO_2_ (per 5 %)	1.05	1.02–1.09	0.002
Hypocapnia	1.77	1.32–2.39	<0.001
Hypercapnia	1.08	0.63–1.81	0.774
Acidemia	1.77	1.26–2.47	<0.001
Alkalemia	1.42	0.99–2.03	0.055

BMI: Body mass index; CI: Confidence interval; BUN: Blood urea nitrogen; FiO_2_: Inspired oxygen fraction; MAP: Mean arterial pressure; SpO_2_: Peripheral oxygen saturation; VR: Ventilatory ratio.

### Model performance

**Figure 2:**
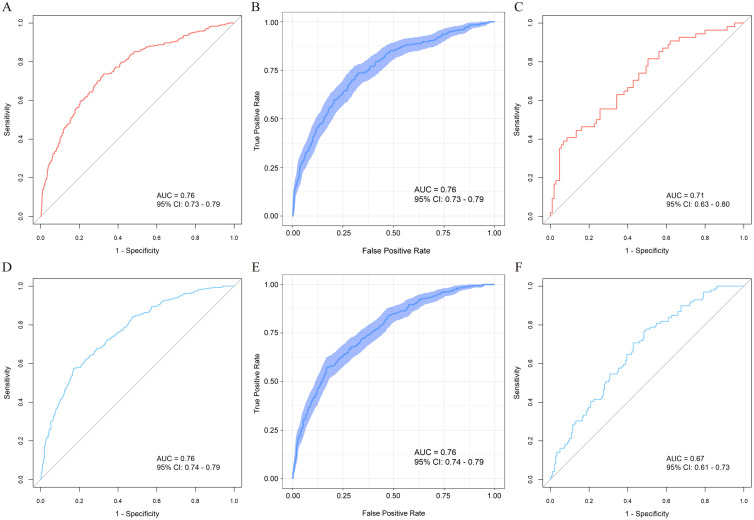
ROC curves of our models. A: Training cohort of high VR model. B: Internal validation of high VR model. C: External validation cohort of high VR model. D: Training cohort of low VR model. E: Internal validation of low VR model. F: External validation cohort of low VR model. AUC: The area under the curve; CI: Confidence interval; ROC: Receiver operating characteristic; VR: Ventilatory ratio.

Model discrimination assessment revealed that both high and low VR subgroup models demonstrated moderate discriminatory capability. In model training set, high VR model achieved an area under the curve (AUC) of 0.76 (95 % CI: 0.73 to 0.79; Figure 2A). High VR model remained moderately discriminating in internal validation (AUC: 0.76, 95 % CI: 0.73 to 0.79; Figure 2B) and external validation (AUC: 0.71, 95 % CI: 0.63 to 0.80; Figure 2C). For low VR model, the AUC within the training set was 0.76 (95 % CI: 0.74 to 0.79; Figure 2D). Low VR model also maintained moderate discriminatory ability in internal validation (AUC: 0.76, 95 % CI: 0.74–0.79; Figure 2E) and external validation (AUC: 0.67, 95 % CI: 0.61 to 0.73; Figure 2F).

**Figure 3:**
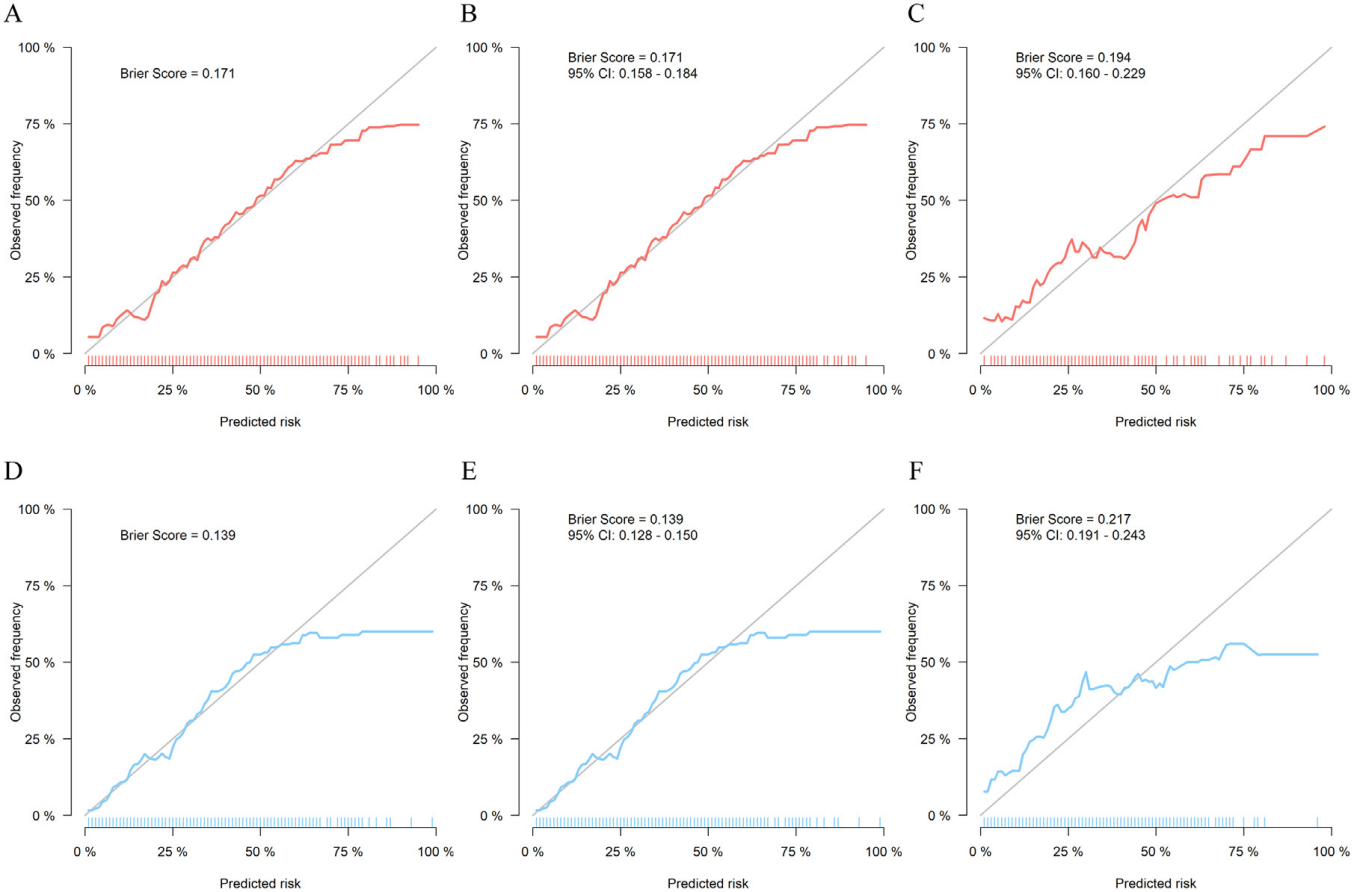
Calibration of our models. A: Training cohort of high VR model. B: Internal validation of high VR model. C:External validation cohort of high VR model. D: Training cohort of low VR model. E: Internal validation of low VR model.F: External validation cohort of low VR model. CI: Confidence interval; VR: Ventilatory ratio.

Regarding calibration, the Brier scores for both models ranged between 0 and 0.25, indicating good calibration of the prediction models. Specifically, in the training set, the Brier score for the high VR model was 0.171 (Figure 3A), and the Brier score in bootstrap internal validation and external validation were 0.171 (95 % CI: 0.158 to 0.184; Figure 3B) and 0.194 (95 % CI: 0.160 to 0.229; Figure 3C), respectively. For the low VR model, the Brier score was 0.139 (Figure 3D) in the training set and 0.139 (95 % CI: 0.128 to 0.150; Figure 3E) in internal validation, and 0.217 (95 % CI: 0.191 to 0.243; Figure 3F) in external validation.

**Figure 4:**
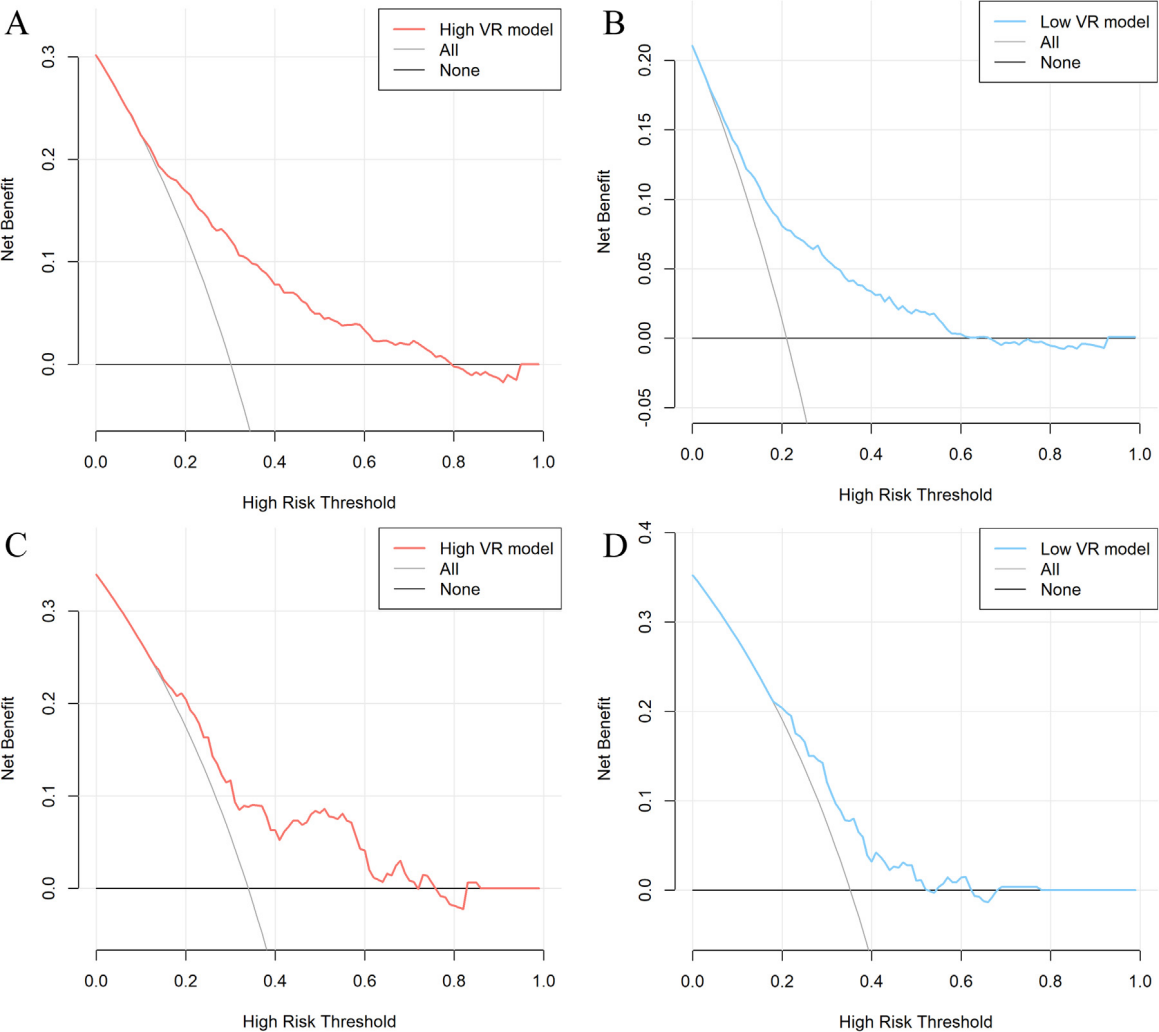
Decision curve analysis of our models. A: Training cohort of high VR model; B: Training cohort of low VR model; C: External validation cohort of high VR model; D: External validation cohort of low VR model. VR: Ventilatory ratio.

For assessing clinical utility, DCA demonstrated that the DCA curve for the high VR model was positioned away from the two extreme curves across a threshold probability range of 0.2 to 0.8 (Figure 4A), and the DCA curve for the low VR model was positioned away from the two extreme curves across a threshold probability range of 0.1 to 0.6 (Figure 4B), indicating reasonable clinical utility. External validation further demonstrated that the DCA curve for the high VR model remained separated from the extreme curves within a threshold probability range of 0.2 to 0.6 (Figure 4C), and the DCA curve for the low VR model remained separated from the extreme curves within a threshold probability range of 0.2 to 0.5 (Figure 4D), suggesting continued clinical utility in the external validation set.

### Variable importance evaluation and model cross-prediction

Variable relative importance was quantified by subtracting the predictor’s degrees of freedom from the variable’s Wald chi-squared statistic. The results indicated that for the high VR model, the top five variables, ranked in descending order of importance, were: age, BUN, peak pressure, minute ventilation, and heart rate. For the low VR model, the top five variables, also ranked in descending order of importance, were: age, heart rate, hypocapnia, bilirubin, and peak pressure (Supplementary Figure S5). Supplementary Figure S6 illustrates the overlap and distinctness of predictor variables incorporated into the high VR and low VR models. Specifically, age, body temperature, heart rate, peak pressure, fraction of inspired oxygen, BUN, and total bilirubin were common predictor variables in the prognostic models for both the high and low VR groups. Minute ventilation, MAP, BMI and SpO_2_ were unique predictor variables in the high VR model, whereas hypocapnia, acidemia, respiratory rate, and platelet count were unique predictor variables in the low VR model.

**Figure 5:**
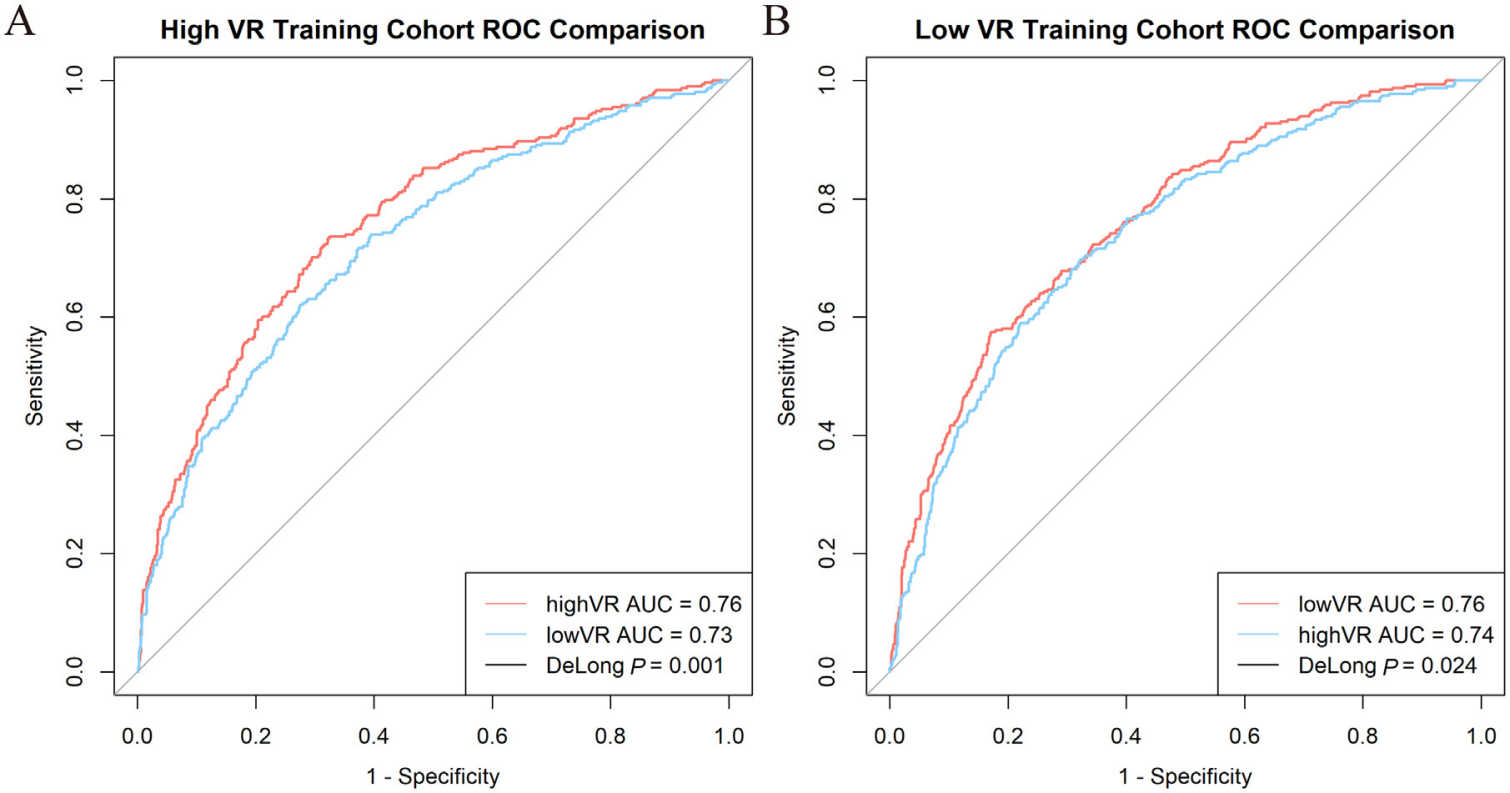
Comparison of ROC curves between high VR model and low VR model in the same cohort. A: High VR training cohort; B: Low VR training cohort. AUC: The area under the curve; ROC: receiver operating characteristic; VR: Ventilatory ratio.

To assess the performance disparity between the high VR model and the low VR model within the same ventilation ratio subgroup, this study employed the two prognostic prediction models, developed in the high VR population and the low VR population, respectively, to conduct predictions across both model derivation cohorts. The difference in discrimination between the two models within the same subgroup population was then compared. The analysis results, presented in Figure 5, indicated that the AUC of the high VR model when predicting prognosis in the low VR subgroup population was 0.74, significantly lower than that of the low VR model (DeLong test; *P*=0.024). Furthermore, the AUC of the low VR model when predicting 30-day mortality in the high VR subgroup population was 0.73, significantly lower than that of the high VR model (DeLong test; *P*=0.001).

## Discussion

This study developed and validated distinct 30-day mortality prediction models for ARDS patients stratified by VR, demonstrating good discrimination, calibration, and clinical utility across both internal and external cohorts. Importantly, the models incorporated routinely available bedside variables, balancing simplicity with performance. Differences in predictor sets and model performance between high and low VR subgroups highlight the physiological heterogeneity in ARDS and support the need for subgroup-specific prognostic tools.

The VR is a practical surrogate for dead space fraction and reflects ventilatory inefficiency in ARDS^[^^15^^]^ Prior studies, including Sinha et al.,^[^[16], [18]^]^ have shown that a baseline VR ≥ 2 is associated with significantly higher mortality. From a physiological perspective, elevated VR indicates increased dead space and impaired CO_2_ clearance, often due to overdistended or non-perfused lung regions.^[^[15], [19]^]^ In our cohort, VR subgroup assignments were stable over the first 24 h, with 89.8 % of patients in the high VR group and 94.2 % in the low VR group retaining their classification from Day 0 to Day 1. This short-term stability, consistent with Delucchi et al’s^[^^20^^]^ findings on ARDS subphenotypes, suggests that VR-defined groups represent persistent physiological states suitable for targeted risk stratification.

The prognostic models developed for the high and low VR subgroups differed in their included risk factors, suggesting that mortality determinants vary by VR level. The observed variation in predictive performance across these subgroup-specific models highlights the need for stratified management in ARDS. This supports the use of tailored prediction models over a one-size-fits-all approach. Consistent with our findings, Obermeyer et al.^[^^21^^]^ demonstrated that ignoring subgroup heterogeneity can lead to significant performance loss due to variable distribution differences across populations.

This study identified several shared prognostic factors across both VR subgroups, including age, heart rate, peak inspiratory pressure, FiO_2_, BUN, and total bilirubin, variables consistently linked to poor outcomes in previous ARDS studies.^[^[22], [23], [24], [25]^]^ In contrast, higher body temperature was associated with improved prognosis.^[^^26^^]^ Additional predictors like platelet count, SpO₂, and MAP also aligned with established risk models.^[^^23^^,^^27^^,^^28^^]^ Importantly, minute ventilation and respiratory rate emerged as subgroup-specific predictors in the high and low VR models, respectively. As components of mechanical power, both are implicated in ventilator-induced lung injury (VILI).^[^[29], [30], [31]^]^ In high VR subgroup, elevated VR primarily indicates a significant increase in dead space ventilation and reduced efficiency in carbon dioxide clearance.^[^^15^^]^ To compensate for this inefficiency and maintain adequate CO_2_ removal, patients might require higher minute ventilation.^[^^32^^]^ While necessary for gas exchange, excessively high minute ventilation contributes substantially to mechanical power, thereby increasing the risk of VILI.^[^^31^^]^ The predictive importance of minute ventilation in the high VR model highlights the balance between optimizing CO_2_ clearance and VILI from excessive mechanical power. In low VR subgroup, patients exhibit relatively efficient ventilation and less prominent dead space. Although minute ventilation is still relevant, respiratory rate emerged as a distinct predictor. An elevated respiratory rate, even with relatively preserved ventilatory efficiency, can indicate increased respiratory drive, heightened metabolic demand, or an attempt to compensate for other physiological disturbances.^[^^32^^,^^33^^]^ Thus, in low VR patients, close monitoring and management of respiratory rate become particularly important to mitigate the risk of VILI, suggesting that controlling the frequency of breaths may be a more sensitive lever for lung protection in this specific phenotype.

Additionally, hypocapnia was independently associated with increased mortality in the low VR subgroup, consistent with the secondary analysis of the LUNG SAFE study.^[^^34^^]^ Hypocapnia may worsen lung injury through increased permeability, edema, reduced compliance, and inflammation.^[^[35], [36], [37]^]^ Importantly, hypocapnia is often achieved through high tidal volume ventilation, which itself directly causes VILI, contributing to higher mortality in ARDS patients.^[^^34^^]^ The LUNG SAFE study also found that lower arterial pH was linked to worse ARDS outcomes^[^^22^^]^ suggesting that elevated pH acts as a protective factor for ARDS prognosis. However, its analysis assumed a linear relationship, overlooking potential non-linear effects. Other studies have shown that alkalemia may also increase mortality in critically ill patients.^[^^38^^]^ In our low VR subgroup, acidemia was significantly associated with higher 30-day mortality, while alkalemia showed a borderline association. These findings highlight the importance of maintaining arterial pH and PaCO_2_ within the normal range in low VR ARDS patients.

This study developed and validated subgroup-specific prognostic models based on VR-defined phenotypes and evaluated their generalizability using external cohorts. While our findings support the value of stratified risk assessment in ARDS, several limitations should be acknowledged. First, as a retrospective model development study, causal relationships cannot be inferred. Second, we did not assess interaction effects between VR and individual predictors; thus, potential differential associations (e.g., for minute ventilation or pH) across subgroups remain unexplored. Third, although machine learning was used, model selection was guided by conventional statistical methods, so variables excluded from the final models may still hold prognostic value. Fourth, VR stability was assessed only within the first 24 h; longer-term trends were not analyzed and may influence subgroup classification over time. Last but not least, the retrospective nature of this study, relying on public databases, introduces inherent limitations such as selection bias, missing follow-up data, and potential confounders. Future work could incorporate diverse cohorts (e.g., HIRID or AmsterdamUMCdb), longitudinal VR measurements, and broader clinical variables to enhance the robustness and clinical utility of ARDS prognostic models.

## Conclusions

Two risk models based on routine data moderately predict mortality in ARDS patients with different VR, highlighting subgroup-specific risk factors and suggesting that clinical management of different VR-defined subgroups should have different emphasis. Further evaluation of our models is needed.

## CRediT authorship contribution statement

**Zhangwei Liang:** Writing – review & editing, Writing – original draft, Formal analysis. **Xinyi Luo:** Writing – original draft, Data curation. **Ya Wang:** Writing – review & editing. **Weilin Wang:** Formal analysis. **Yuelin Liu:** Data curation. **Ying Zhu:** Data curation. **Juan Ouyang:** Data curation. **Jie Zhang:** Supervision. **Yin Xi:** Supervision. **Yimin Li:** Supervision, Conceptualization. **Yonghao Xu:** Supervision, Conceptualization.

## Appendix Supplementary materials

Image, application 1
