# Supplementary material for: Clinical models for predicting 30-day mortality in ARDS: A focus on ventilatory ratio-defined subgroups
Source: J Intensive Med. 2025 Dec 8;6(2):185–95. doi: 10.1016/j.jointm.2025.09.002 (PMC13100851; doi:10.1016/j.jointm.2025.09.002)
Supplement: Supplementary file 1 [file mmc1.docx]

# Supplemental Material

Supplementary Method

*Study Period and ARDS Diagnostic Criteria*

Patients were enrolled from various sources, each with specific inclusion periods and ARDS diagnostic criteria. For the Medical Information Mart for Intensive Care (MIMIC-IV) and eICU Collaborative Research Database (eICU-CRD), patients diagnosed with ARDS were included from 2014 to 2019 and 2014 to 2015 respectively. Due to date offsetting in the eICU database for privacy reasons, the precise month and day for the start and end of patient inclusion in both MIMIC-IV and eICU-CRD cannot be specified. For the National Heart, Lung, and Blood Institute (NHLBI)-sponsored ARDS Network randomized controlled trials, the recruitment periods were as follows: ARMA from March 1996 through March 1999, KARMA from March 1996 through March 1997, LARMA from February 1998 through May 1999, ALVEOLI from October 1999 through February 2002, FACCT from June 2000 through October 2005, and ALTA from August 2007 through November 2008.

Regarding ARDS diagnostic criteria, patients from the ARMA/LARMA/KARMA, ALVEOLI, FACCT, and ALTA studies utilized the American-European Consensus Conference definition for ARDS^[1]^. However, it is important to note that patients were enrolled in these studies if they met the diagnostic criteria for Acute Lung Injury (ALI) at the time of their inclusion. In contrast, for the MIMIC-IV and eICU-CRD databases, ARDS diagnosis adhered to the Berlin definition of ARDS^[2]^.

Table S1. Baseline and clinical characteristics of patients of external validation cohorts.

|  | Low VR external validation cohort (*n*=281) | High VR external validation cohort (*n*=159) |
| --- | --- | --- |
| Age (years) | 62.00(51.00,73.00) | 56.00(45.00,66.00) |
| Sex(Female) | 92(32.7) | 77(48.4) |
| Ethnicity |  |  |
| White | 161(57.7) | 89(56.0) |
| Black | 20(7.2) | 9(5.7) |
| Other | 98(35.1) | 61(38.4) |
| ARDS risk factors |  |  |
| Pneumonia | 70(41.7) | 57(49.6) |
| Sepsis | 37(22.0) | 16(13.9) |
| Aspiration | 23(13.7) | 22(19.1) |
| Trauma | 3(1.8) | 2(1.7) |
| Other | 35(20.8) | 18(15.7) |
| BMI (kg/m^2^) | 28.34(23.52,33.64) | 31.16(25.90,37.30) |
| Heart rate (beats/min) | 98.51(22.50) | 102.00(24.95) |
| MAP (mmHg) | 76.00(66.00,93.00) | 76.00(69.00,87.00) |
| Body temperature (℃) | 37.10(0.85) | 36.90(1.21) |
| SpO_2_ (%) | 98.00(95.00,100.00) | 95.00(90.00,98.00) |
| White blood cell (×10^9^/L) | 13.60(9.20,19.20) | 13.45(8.97,20.45) |
| HCT (%) | 31.70(26.40,36.90) | 35.10(28.65,40.60) |
| Platelets (×10^9^/L) | 154.00(93.00,224.00) | 178.00(115.50,239.50) |
| Creatinine (mg/dL) | 1.31(0.80,2.15) | 1.30(0.91,2.00) |
| BUN (mg/dL) | 27.00(18.00,46.00) | 25.00(15.00,36.50) |
| Bilirubin (mg/dL) | 0.80(0.50,2.20) | 0.80(0.40,1.60) |
| Tidal volume/PBW (mL/kg) | 6.74(6.10,7.78) | 7.32(6.42,8.88) |
| Respiratory rate (breaths/min) | 20.00(16.00,24.00) | 24.00(20.00,29.00) |
| Minute ventilation (L/min) | 8.40(7.10,10.40) | 11.90(8.84,13.65) |
| PEEP (cmH_2_O) | 5.90(5.00,10.00) | 10.00(5.00,12.00) |
| Plateau pressure (cmH_2_O) | 20.00(17.00,24.00) | 24.00(21.00,28.00) |
| Peak inspiratory pressure (cmH_2_O) | 23.00(19.00,28.00) | 28.00(23.50,33.00) |
| Driving pressure (cmH_2_O) | 12.25(10.00,15.00) | 14.00(11.00,18.10) |
| Compliance (mL/cmH_2_O) | 37.00(27.86,47.10) | 29.71(22.99,41.88) |
| Ventilatory ratio | 1.47(1.18,1.71) | 2.54(2.22,2.99) |
| FiO_2_ (%) | 60.00(50.00,100.00) | 80.00(50.00,100.00) |
| Arterial PaO_2_ (mmHg) | 90.00(69.00,132.00) | 78.00(58.00,102.00) |
| Arterial PaO_2_/FiO_2_ ratio (mmHg) | 143.75(99.00,197.50) | 105.00(71.43,147.67) |
| Arterial PaCO_2_ (mmHg) | 39.00(34.00,45.50) | 53.00(44.45,63.50) |
| Arterial pH | 7.34(7.26,7.40) | 7.24(7.15,7.33) |
| Bicarbonate (mmol/L) | 21.00(17.00,23.00) | 22.00(18.00,26.00) |
| 30-day mortality (%) | 99(35.2) | 54(34.0) |

BMI: Body Mass Index, BUN: Blood Urea Nitrogen, HCT: Hematocrit, MAP: Mean Arterial Pressure, PEEP: Positive End-Expiratory Pressure.

Table S2. Transitions between VR classes from day 0 to day 1 in ARDSNet cohort.

|  |  | Day 1 | |
| --- | --- | --- | --- |
|  |  | High-VR | Low-VR |
| Day 0 | High-VR | 694(89.8) | 79(10.2) |
|  | Low-VR | 62(5.8) | 1012(94.2) |

Table represents transition probabilities for each class at day 1 compared with day 0; numbers in parentheses are numbers of patients in each group.

Table S3. Univariable Logistic Regression of Clinically Relevant Variables in High VR Patients.

| Variable | OR | 95% CI | *P* |
| --- | --- | --- | --- |
| Sex (Female) | 0.82 | 0.62-1.06 | 0.133 |
| Age | 1.04 | 1.03-1.05 | <0.001 |
| BMI | 0.97 | 0.96-0.99 | 0.003 |
| Heart rate, per 10 beats/min | 1.07 | 1.00-1.15 | 0.039 |
| MAP, per 10mmHg | 0.83 | 0.75-0.91 | <0.001 |
| Body temperature | 0.87 | 0.75-1.00 | 0.047 |
| SpO_2_ | 0.93 | 0.91-0.96 | <0.001 |
| White blood cell | 1.01 | 0.99-1.02 | 0.384 |
| HCT | 0.99 | 0.97-1.01 | 0.271 |
| Platelets, per 10×10^9^/L | 0.99 | 0.98-1.00 | 0.038 |
| Creatinine | 1.16 | 1.06-1.27 | 0.002 |
| BUN | 1.02 | 1.02-1.03 | <0.001 |
| Bilirubin | 1.07 | 1.02-1.13 | 0.009 |
| Tidal volume/PBW, mL/kg | 1.03 | 0.97-1.09 | 0.304 |
| Respiratory rate, per 5 breaths/min | 1.09 | 1.00-1.20 | 0.05 |
| Minute ventilation | 1.07 | 1.04-1.11 | <0.001 |
| PEEP | 1.03 | 1.00-1.07 | 0.038 |
| Plateau pressure | 1.02 | 1.01-1.04 | 0.012 |
| Peak inspiratory pressure | 1.02 | 1.01-1.03 | 0.001 |
| Driving pressure | 1.01 | 1.00-1.03 | 0.121 |
| FiO_2_, per 5% | 1.09 | 1.05-1.13 | <0.001 |
| PaO_2_/FiO_2_, per 10mmHg | 0.96 | 0.94-0.99 | 0.002 |
| Bicarbonate | 0.95 | 0.93-0.98 | <0.001 |
| Hypocapnia | 1.60 | 1.14-2.25 | 0.006 |
| Hypercapnia | 1.01 | 0.73-1.39 | 0.947 |
| Acidemia | 1.35 | 1.02-1.78 | 0.037 |
| Alkalemia | 0.73 | 0.44-1.19 | 0.223 |

BMI: Body Mass Index, BUN: Blood Urea Nitrogen, HCT: Hematocrit, MAP: Mean Arterial Pressure, PEEP: Positive End-Expiratory Pressure.

Table S4. Univariable Logistic Regression of Clinically Relevant Variables in Low VR Patients.

| Variable | OR | 95% CI | *P* |
| --- | --- | --- | --- |
| Sex(Female) | 0.86 | 0.66-1.11 | 0.236 |
| Age | 1.03 | 1.02-1.04 | <0.001 |
| BMI | 0.98 | 0.96-1.00 | 0.033 |
| Heart rate, per 10 beats/min | 1.12 | 1.06-1.19 | <0.001 |
| MAP, per 10mmHg | 0.86 | 0.78-0.94 | 0.001 |
| Body temperature | 0.86 | 0.76-0.97 | 0.015 |
| SpO_2_ | 0.95 | 0.92-0.98 | 0.003 |
| White blood cell | 0.99 | 0.97-1.00 | 0.045 |
| HCT | 0.99 | 0.97-1.01 | 0.229 |
| Platelets, per 10×10^9^/L | 0.98 | 0.97-0.99 | <0.001 |
| Creatinine | 1.14 | 1.05-1.23 | 0.001 |
| BUN | 1.02 | 1.01-1.02 | <0.001 |
| Bilirubin | 1.09 | 1.05-1.13 | <0.001 |
| Tidal volume/PBW, mL/kg | 1.08 | 1.02-1.15 | 0.009 |
| Respiratory rate, per 5 breaths/min | 1.20 | 1.10-1.31 | <0.001 |
| Minute ventilation | 1.11 | 1.06-1.15 | <0.001 |
| PEEP | 1.01 | 0.97-1.04 | 0.700 |
| Plateau pressure | 1.02 | 1.00-1.04 | 0.033 |
| Peak inspiratory pressure | 1.02 | 1.00-1.03 | 0.017 |
| Driving pressure | 1.02 | 1.00-1.04 | 0.032 |
| FiO_2_, per 5% | 1.06 | 1.03-1.09 | <0.001 |
| PaO_2_/FiO_2_, per 10mmHg | 0.97 | 0.95-0.99 | 0.001 |
| Bicarbonate | 0.92 | 0.90-0.94 | <0.001 |
| Hypocapnia | 1.91 | 1.47-2.49 | <0.001 |
| Hypercapnia | 1.05 | 0.66-1.68 | 0.823 |
| Acidemia | 1.97 | 1.47-2.65 | <0.001 |
| Alkalemia | 1.19 | 0.86-1.64 | 0.290 |

BMI: Body Mass Index, BUN: Blood Urea Nitrogen, HCT: Hematocrit, MAP: Mean Arterial Pressure, PEEP: Positive End-Expiratory Pressure.


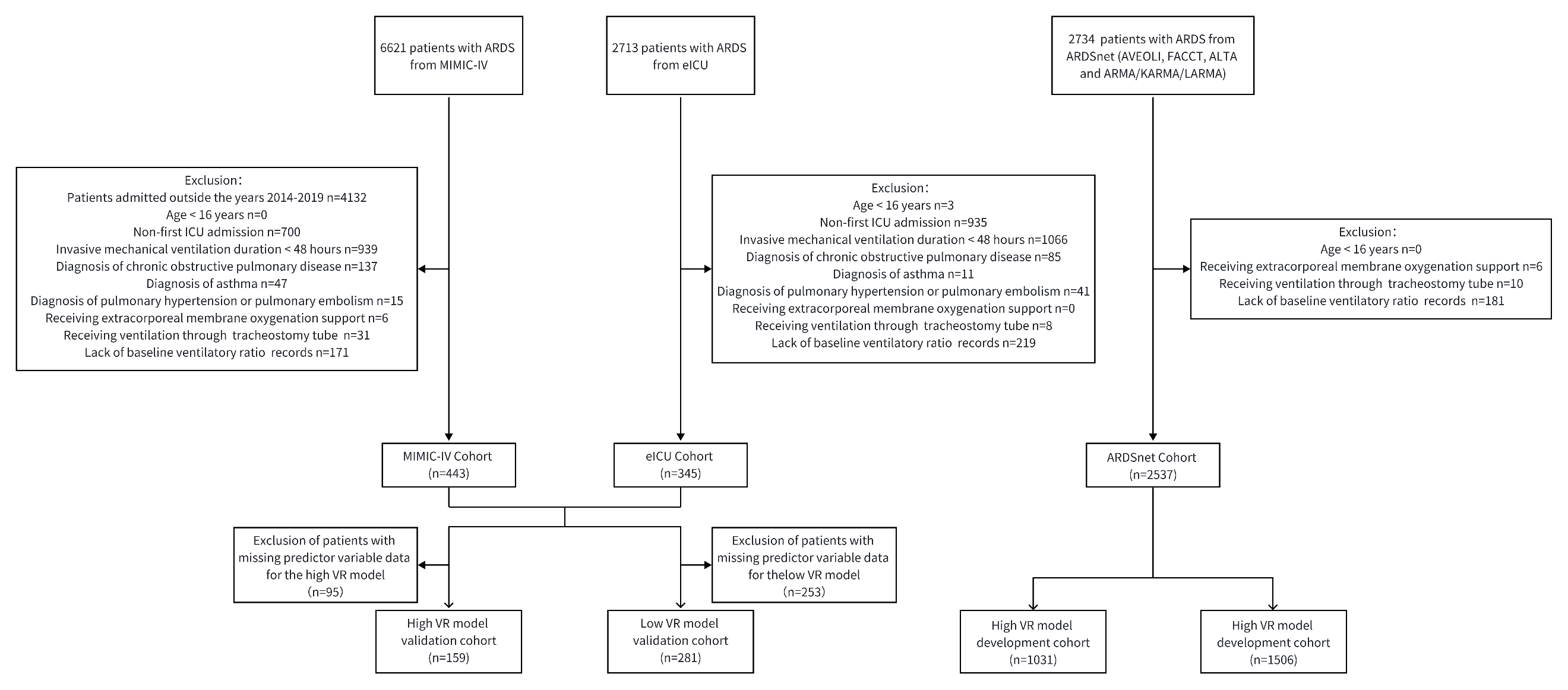


Figure S1. Flowchart of inclusion and exclusion.

Figure S2. Multicollinearity test using Variance Inflation Factor.

(a)High VR model; (b)Low VR model. A1: Age, A2: Bilirubin, A3: Body Mass Index, A4: Blood Urea Nitrogen, A5:FiO_2_, A6: Heart rate, A7: Mean Arterial Pressure, A8: Minute ventilation, A9: Peak pressure, A10: SpO_2_, A11: Body temperature, B1: Age, B2: Bilirubin, B3: Blood Urea Nitrogen, B4: FiO_2_, B5: Heart rate, B6: Arterial PaCO_2_, B7: Peak pressure, B8: Arterial pH, B9: Platelets, B10: Respiratory rate, B11: Body temperature. B6 was categorized into hypocapnia, hypercapnia and normal(reference). B8 was categorized into acidemia, alkalemia and normal(reference).


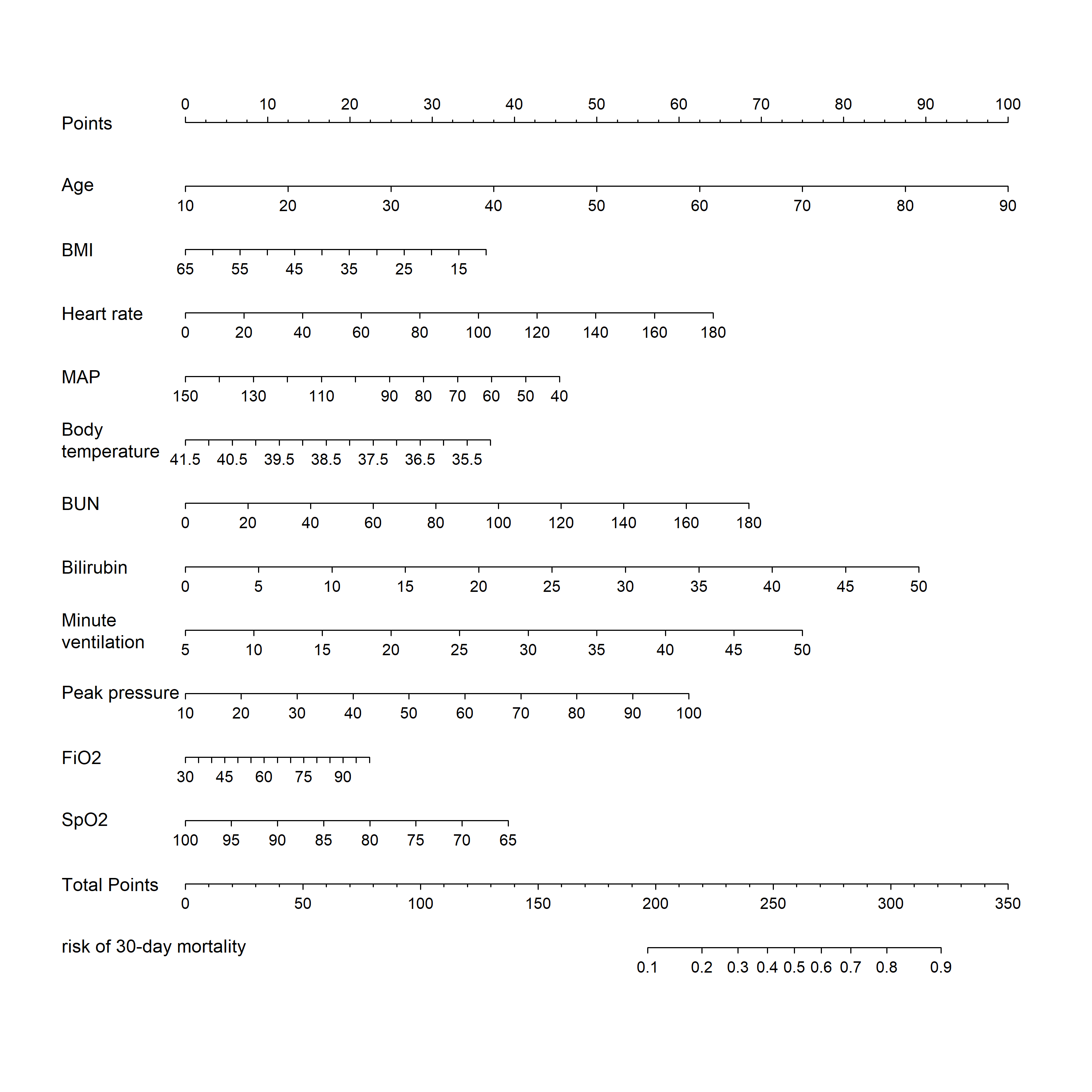


**Figure S3**. Nomogram of High VR model to estimate the risk of 30-day mortality. BMI: Body Mass Index, BUN: Blood Urea Nitrogen, MAP: Mean Arterial Pressure.


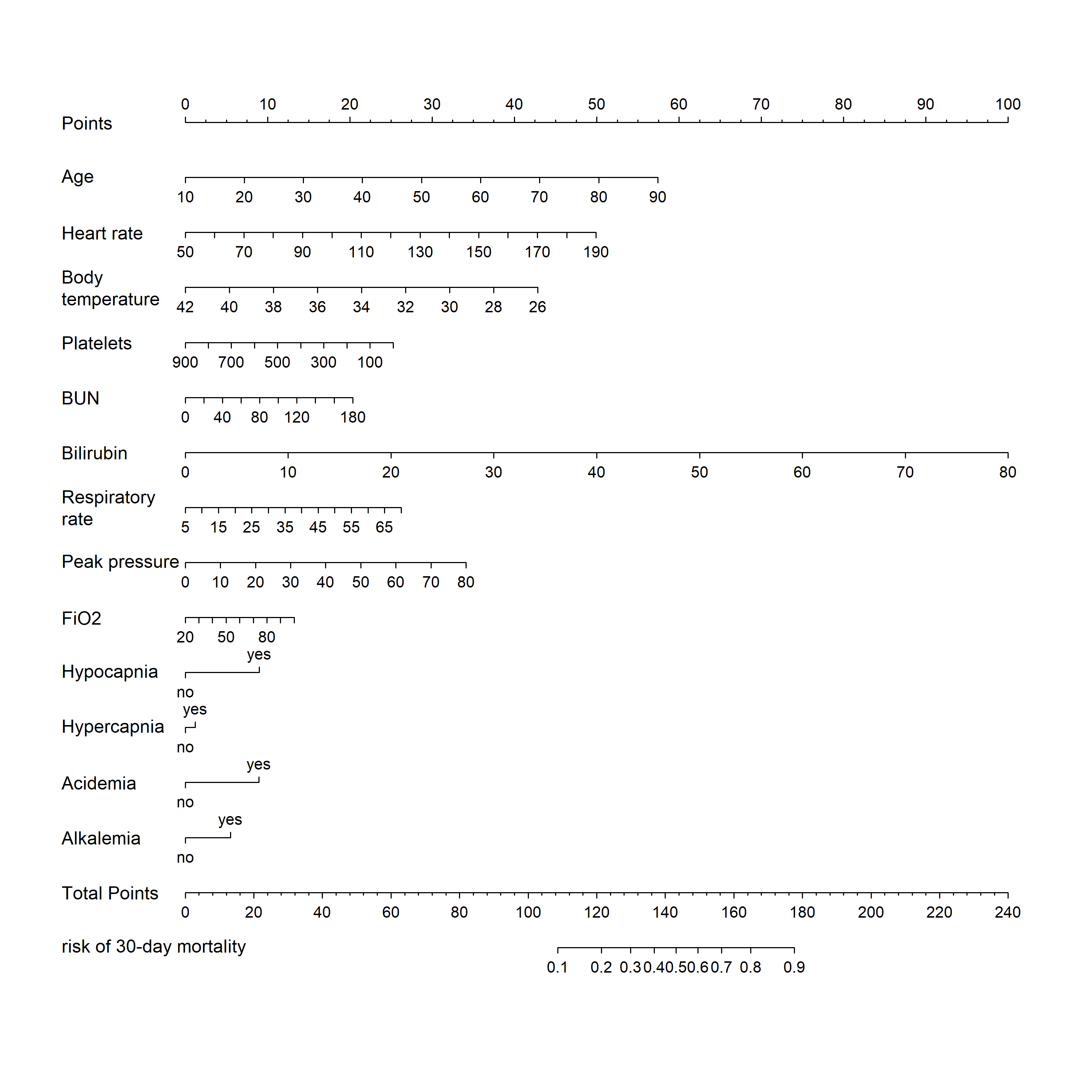


**Figure S4**. Nomogram of Low VR model to estimate the risk of 30-day mortality. BUN: Blood Urea Nitrogen.


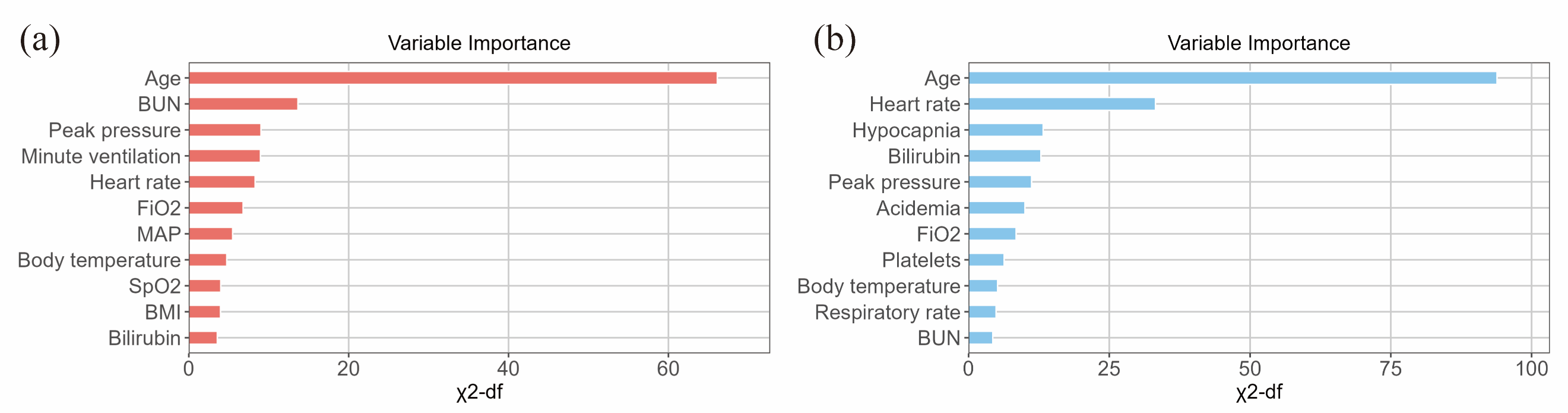


**Figure S5**. Importance of variables included in the predictive models. The importance of each variable is measured as the chi-square statistic minus the predicted degrees of freedom. **a** High VR model; **b** Low VR model; BMI: Body Mass Index, BUN: Blood Urea Nitrogen, MAP: Mean Arterial Pressure.


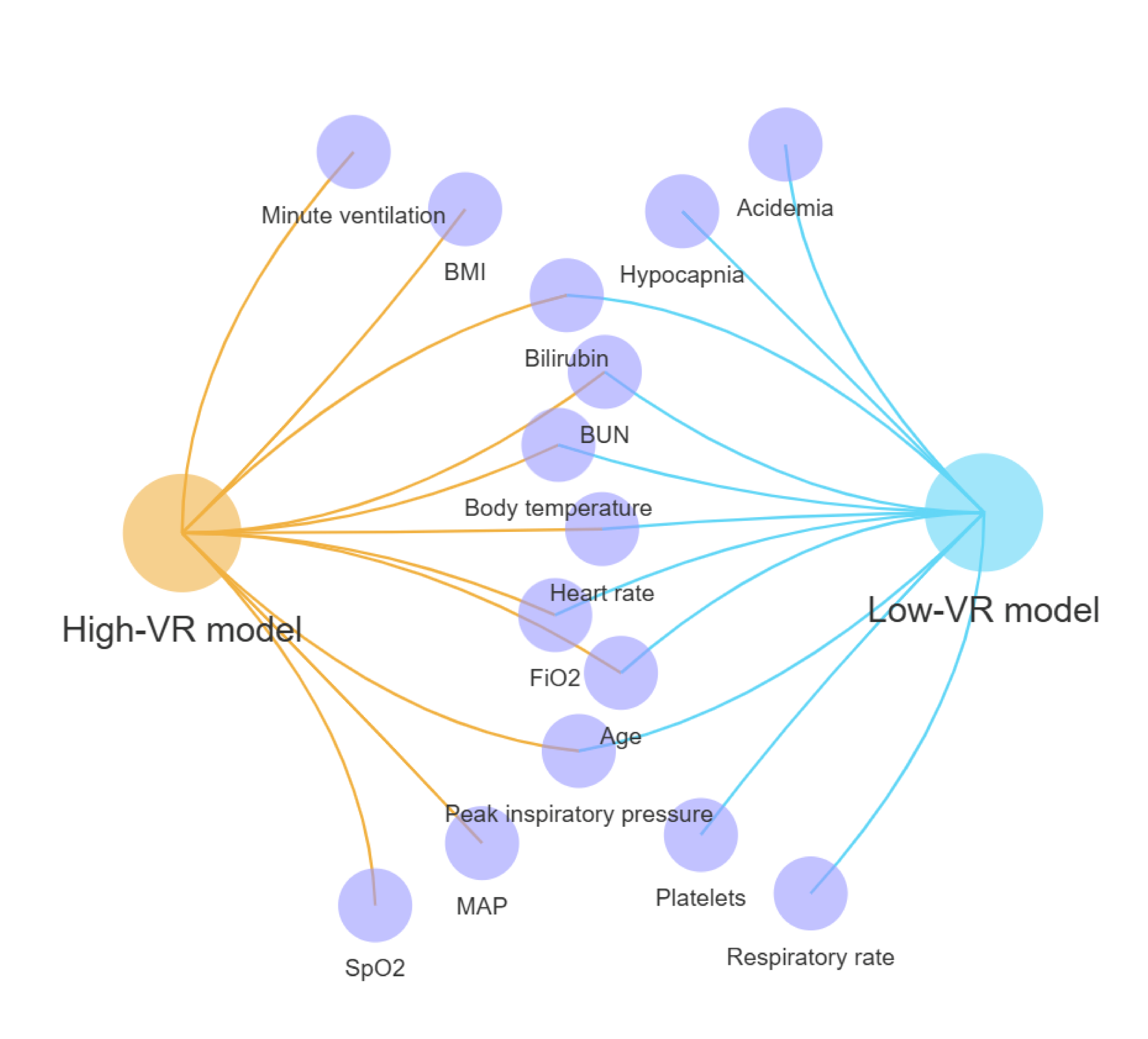


**Figure S6**. Venn diagram of the variables of high VR model versus low VR model.

References

[1] Bernard GR, Artigas A, Brigham KL, Carlet J, Falke K, Hudson L, et al. The American-European Consensus Conference on ARDS. Definitions, mechanisms, relevant outcomes, and clinical trial coordination. American journal of respiratory and critical care medicine 1994;149(3 Pt 1):818-24. doi: 10.1164/ajrccm.149.3.7509706.

[2] Ranieri VM, Rubenfeld GD, Thompson BT, Ferguson ND, Caldwell E, Fan E, et al. Acute respiratory distress syndrome: the Berlin Definition. Jama 2012;307(23):2526-33. doi: 10.1001/jama.2012.5669.
